# Informing efforts beyond tailored promotional campaigns by understanding contextual factors shaping vaccine hesitancy among equity-deserving populations in Canada: an exploratory qualitative study

**DOI:** 10.1186/s12939-023-02025-y

**Published:** 2023-10-07

**Authors:** Lena G. Nascimento, Ève Dubé, Kathleen E. Burns, Patrick Brown, Michael Calnan, Paul R. Ward, Eric Filice, Hoda Herati, Nnenna A. U. Ike, Bobbi Rotolo, Samantha B. Meyer

**Affiliations:** 1 University of Waterloo, Waterloo, Canada; 2 Institut National de Santé Publique du Québec, Québec City, Canada; 3 University of Amsterdam, Amsterdam, The Netherlands; 4 University of Kent, Canterbury, UK; 5 Torrens University, Adelaide, Australia; 6 Unity Health Toronto, Toronto, Canada

**Keywords:** COVID-19, Equity-deserving groups, Marginalized groups, Canada, Vaccine hesitancy, Contextual factors, Promotional campaigns, Vaccine confidence, Qualitative

## Abstract

**Background:**

Vaccine hesitancy exists on a continuum ranging between complete adherence and complete refusal due to doubts or concerns within a heterogeneous group of individuals. Despite widespread acknowledgement of the contextual factors influencing attitudes and beliefs shaping COVID-19 vaccine hesitancy, qualitative research with equity-deserving groups, accounting for unique lived experiences, remains a gap in the literature. We aim to identify and begin to understand and document the unique contextual factors shaping hesitancy by equity-deserving groups as it relates to relationships with government and health authorities.

**Methods:**

Participants were recruited and interviewed between Aug-Dec 2021. Semi-structured interviews using a convergent interviewing technique were conducted with individuals from the general population, as well as individuals who identify as First Nations, Métis, or Inuit, members of the LGBT2SQ + community, low-income Canadians, Black Canadians, and newcomers. Interviews were audio recorded and transcribed by a team of researchers. Memos were written following interviews and used to complement the thematic analysis of the interview data. Themes are presented in the results section.

**Results:**

The rationale for hesitancy among equity-deserving groups is consistent with literature documenting hesitancy in the general population. Contextual factors surrounding equity-deserving groups’ attitudes and beliefs, however, are unique and relate to a history of oppression, discrimination, and genocide. We identified factors unique to subgroups; for example, religious or fatalistic beliefs among participant who identify as FNMI, fear associated with lack of testing and speed of vaccines’ production among participants who identify as FNMI, Black, and LGBT2SQ + , distrust of the healthcare system for LGBT2SQ + and Black Canadians, and distrust of the government and opposition to vaccine mandates for participating who identify as LGBT2SQ + , low-income, FNMI, or Black Canadian. Newcomers stood out as very trusting of the government and accepting of COVID-19 vaccination.

**Conclusions:**

While our data on vaccine hesitancy largely mirror concerns reported in the vast body of literature citing rationale for COVID-19 hesitancy in high-income countries, the contextual factors identified in our work point to the need for wider systemic change. Our results may be used to support efforts, beyond tailored promotion campaigns, to support the confident acceptance of vaccines for COVID-19 and the acceptance of novel vaccines as future infectious diseases emerge.

## Background

Canada’s first COVID-19 case was confirmed on January 23, 2020, in Toronto, Ontario [1, 2]. In response, government officials called upon the public to trust and accept measures to mitigate the risk of COVID-19 spread. One such measure was vaccination, the uptake of which is complex and multifactorial [3]. Vaccine hesitancy (VH), a long-studied phenomenon particularly as it relates to childhood vaccination, became a critical consideration and concern for the management of COVID-19 in Canada and elsewhere, with the World Health Organization (WHO) naming VH one of the top ten threats to global health in 2019 [4]. Within the present paper, we conceptualize VH as existing on a continuum ranging between complete adherence and complete refusal due to doubts or concerns within a heterogeneous group of individuals who may be influenced by a combination of cognitive, emotional, cultural, social, spiritual, and political factors [5–7]. Within these extremes, there is a varying period of delay in acceptance or refusal of vaccination, despite vaccines being readily available to the public [8]. Central to the present work, hesitancy is influenced by the historical, political, and sociocultural contexts in which vaccination occurs [7]. Furthermore, hesitancy is vaccine dependent; for example, there has been criticism that public health messaging around COVID-19 vaccines resembled those aiming at reducing VH for routine immunization and, as a result, did not sufficiently address the constant changes to COVID-19 vaccine recommendations [9]. As such, across the population, attitudes and beliefs regarding vaccination, and thus strategies to promote vaccination, will vary considerably – and even more so given the novelty of the COVID-19 vaccine.

COVID-19 VH in high-income countries is well documented and has been associated with a number of factors, including the absence of a recent history of influenza vaccination, lower perceived risk of contracting COVID-19, reduced fear of COVID-19 disease, lower perceived COVID-19 disease severity, absence of a chronic medical condition, belief that vaccines are not safe/effective, concerns with the speed in which COVID-19 vaccines were developed, exposure to misinformation about COVID-19, and public concerns over the safety of vaccines [10–12]. Alternatively, factors associated with vaccine uptake include motivation to protect oneself or others, trust in government, belief that the vaccine is safe and has a low risk of adverse effects, availability of sufficient information about COVID-19 vaccination, greater perceived risk of COVID-19 to others (but not a risk to oneself), being of older age, and previously receiving an influenza vaccine [13, 14].

Over the last few years, the research community has generated a vast body of literature regarding COVID-19 VH as it relates to equity-deserving groups. Equity-deserving has been defined as “a group of people who, because of systemic discrimination, face barriers that prevent them from having the same access to the resources and opportunities that are available to other members of society, and that are necessary for them to attain just outcomes” [15]. Within Canada, the location of the present study, nonwhite (racialized) individuals have been documented to be less likely than white individuals to receive the COVID-19 vaccine [16]. Being indigenous, black, multiracial or a visible minority has been associated with a lower intention to get vaccinated [16, 17]. This finding is consistent within high-income countries; for example, research has found an association between individuals who identify as Black, Indigenous, BIPOC or as part of the LGBTQ + community and VH [18, 19]. Furthermore, in terms of VH, individuals who immigrated to Canada (compared to Canadian-born individuals) and individuals of lower socioeconomic status have been found to be more hesitant toward COVID-19 vaccines [16, 20].

Despite progress made in understanding contextual factors influencing attitudes and beliefs shaping COVID-19 vaccine VH in equity-deserving groups [20], research to date has predominantly relied on survey-based study models [19]. The present study draws on interviews to understand the lived experiences of vulnerable populations [21] disproportionately impacted by the COVID-19 pandemic [22–26] and whose voices are predominantly absent from public health efforts relating to pandemic preparedness [27]. The need to increase the representation of equity-deserving groups in public health research is necessary not only to inform and drive vaccination efforts with hopes to increase COVID-19 vaccination uptake among equity-deserving groups but also to reduce health disparities and get one step closer toward achieving health equity.

Given that attitudes and beliefs can influence behavior, government and public health have worked to tailor public health messaging to promote vaccine uptake in diverse communities. However, as we will discuss, the unique contextual factors, as they relate to equity-deserving populations’ relationships with government and health authorities in the past, will require interventions that go beyond tailored vaccine promotion efforts. Indeed, typical promotional materials have been criticized as not addressing specific anxieties elicited by the novel vaccines [9], no less among populations whereby VH is influenced by the historical, political, and sociocultural contexts [7]. Herein, we aim to investigate and document demographic and contextual factors that underly VH within equity-deserving populations. We focus on these populations for two reasons: 1. They have been historically marginalized by government organizations, including healthcare services, and thus less likely to trust, and thus accept, government interventions (e.g., see history) ( [28]); and/or 2. They have been disproportionately impacted by COVID-19 in Canada [29], rendering vaccination critical for health equity [30]. Our results may be used to support efforts, beyond tailored promotion campaigns, to support the confident acceptance of vaccines for COVID-19 but perhaps more importantly, the acceptance of novel vaccines as future infectious diseases emerge.

## Methods

The data presented herein are part of a larger research project investigating the acceptance of COVID-19 countermeasures in Canada. Here, we specifically focus on data collected from participants who self-identified as belonging to an equity-deserving group regarding their perspectives on COVID-19 vaccination.

Semi-structured interviews (*n* = 56) were conducted with individuals aged 18 + between August and December 2021. In addition to the general population (*n* = 19), we specifically sampled subpopulations of equity-deserving populations, including First Nations, Métis, or Inuit (*n* = 7), LGBT2SQ + (*n* = 5), low-income Canadians (less than $40,000 annually; *n* = 8), Black Canadians (*n* = 7), and newcomers (less than 5 years living in Canada; *n* = 10). We acknowledge that each of the subgroups is unique from one another due to differences in shared experiences, cultural beliefs, and practices, which is important given that VH is specific to groups and communities. This, however, does not mean there are no similarities across subgroups. Additionally, we acknowledge that within each of the subgroups, there is diversity across participants, and as such, we do not intend to use our findings for generalization. Participants were recruited through Leger, Canada’s largest and most representative research marketing firm, to gain representation from harder-to-reach populations. Leger recruited potential participants and provided contact information to the research team to obtain consent to participate and schedule the interviews. Participants for our sample size were collected until we reached saturation of themes [31].

We used a convergent interviewing technique [32] via telephone or a virtual platform (Cisco Webex, Zoom or Microsoft Teams), depending on the preference of the participant. Convergent in-depth interviews are characterized by a structured process and unstructured content. Interviews are embedded within a process of design and analysis so that subsequent interviews can build on reflective opportunities from former interviews. This specific interview technique allows for the analysis of interview data to overlap with the collection of that data, and unlike other interview techniques, it is time-efficient, emergent, and data-driven [32]. This approach allowed us to continue data collection until a point where themes were saturated while also ensuring that we explored novel insights relevant to the research aim. For the present paper, we focus on interview questions specifically investigating COVID-19 countermeasures pertaining to vaccination behaviors. Namely, we asked, “Have you been vaccinated against COVID-19?”, “If yes, what was your experience, if no, why not?”, “If periodic boosters end up being recommended (e.g., on a bi, semi or annual basis) do you intend to get them? Why/why not?”, “What are your thoughts about the use of legal mandates by governments to increase vaccination?”, “How has the prospect or actual implementation of vaccine mandates influenced your views on vaccination or your decision to get vaccinated?”. Given the role of contextual factors in VH, participants were also asked to respond to sociodemographic questions and vaccine status. Interviews were conducted by six researchers with the goal of having congruence in social identity between participants and the interviewer, though this was not possible across all participating subgroups.

Interviews were audio recorded and transcribed by an agency abiding by a confidentiality agreement. Following interviews, memos were written by each researcher to document elements of the data meaningful to the project aims. Memos served as a record of the researcher’s initial thoughts on each interview for the purpose of communicating the analytic progress to the team and for recall later down the process of analysis. All six interviewers listened to audio files and prepared memos based on their respective assigned subgroup interviews. Following this procedure, one researcher led the remainder of the analysis, with ongoing input from the team. Initial coding involved staying close to the data and remaining open to exploring all findings relevant to the aim of the interviews. Initial coding, coded by author < removed for blind review > , involved systematically working through the entire dataset, giving full and equal attention to every data point. For this exploratory phase, we were open to coding all data before determining what was or was not meaningful to the analysis [33]. In vivo codes (the participants’ own words) were used to help preserve participants’ meanings of their views and actions. Focused coding involved taking earlier codes that continually reappeared and using them to organize large amounts of the data into meaningful themes and was used to re-examine the initial codes to determine their adequacy and conceptual strength in meeting the research aim. Focused coding was less open-ended and more directed and conceptual, based on themes relevant to their dataset (e.g., coverage of themes relevant to the aim). Within our process of moving from initial to focused coding, we were intentionally attentive to data relevant to the aim of the paper; that is, we were not focused on the most common themes but rather, those that were meaningful.

Ethics approval was obtained by < removed for blind peer review > . All participants provided written or oral consent for the recording and use of quotes in publications. Pseudonyms have been used to maintain anonymity.

## Results

Our results provide timely insight into the sociodemographic factors associated with VH at a time in Canada when there were four COVID-19 vaccines authorized for public use (Pfizer-BioNtech, Moderna, Jessen, and AstraZeneca) and enough vaccine supply for the completion of primary series (1st and 2nd doses) for eligible individuals. The period of data collection also coincided with all provinces and the Yukon territory introducing vaccine passports [34]. Furthermore, information on vaccines’ side effects (common and rare), as well as recommendations/guidelines to populations (i.e., permissible to interchange between authorized COVID-19 vaccines in a two-dose primary series, long-term care residents and seniors living in other congregate settings are recommended to receive their booster dose, and AstraZeneca vaccine use is recommended in younger adults, among others) was also available to the public at this time period [35, 36].

All participants but one selected English (over French) as the primary language. Participants represented all provinces except for NL and NS, with higher representation in ON and AB. Ages ranged from 18–75, with half aged 25–44 years. Most participants were female (*n* = 36), with 19 males and one individual identified as nonbinary. Most participants (*n* = 20) reported earning between $20,000-$59,000.

Table 1 summarizes acceptance of vaccination across subgroups at the time of the interviews. Of all participants (*n* = 56), 51 individuals received their 1st and 2nd doses. Five individuals in total (*n* = 2 Black Canadians, *n* = 1 FNMI and *n* = 2 or low-income) reported nonvaccinated status. Of all participants, eight reported getting the vaccine either because it was mandated or because they felt pressured by their families to receive their vaccine. Two participants expressed a reluctance to accept booster shots or being reluctant to consider the possibility of annual vaccination. Last, at the time the interviews were conducted, no participant had yet received their booster (3^rd^ dose) vaccine.

**Table 1:** Vaccine acceptance by subgroup for participants in the sample

Population Sub-Groups	Have not received the vaccine	Have received one or two doses
Black Canadians (*n* = 7)	2	5
FNMI (*n* = 7)	1	6
LGBT2SQ + (*n* = 5)	0	5
Low-Income (*n* = 8)	2	6
Newcomers (*n* = 10)	0	10
General Population (*n* = 19)	0	19
Total (*n* = 56)	5	51

The following section of this paper showcases findings from our data. Acronyms to categorize equity-deserving groups and the general population were used, such as general population (GP), Black Canadians (BC), LGBT2SQ + (LGBT2SQ +), First Nations, Métis, and Inuit (FNMI), Low Income (LI), and Newcomer (N).

### Vaccine hesitancy across the population

We present data below to underscore that in many ways, the rationale for hesitancy among participants who identify as members of equity-deserving groups is consistent with what is already documented regarding hesitancy across the general population.

Participants explained their own VH (in general and COVID-19 related) in terms of the following key themes:

- Beliefs of resiliency/immunity;
- Vaccination history;
- Low perceived vulnerability based on others’ experiences;
- Perceived risk of severe adverse reactions/vaccine safety (exacerbated by reported adverse reactions);
- Lack of understanding of the vaccine’s mechanisms
- Perceptions of fast development/administration;
- Availability of several brand options of COVID-19 vaccines;
- Perceived lack of need, effectiveness, and purpose for the COVID-19 vaccines;
- Absence/presence of previous vaccination, and
- Lack of trust in the government.

For instance, the following quote demonstrates how participants who reported vaccine acceptance in the past were more accepting of COVID-19 vaccination, suggesting that receiving routine vaccines may be a factor in COVID-19 vaccine acceptance.

*“I didn't really pause and agonize over it a great deal of time. I kept getting vaccines all my life. I went to receive them since I was a child, and I wish I had confidence in them, and I didn't see why they should be any different.” – 75*+*, Woman, LI4*

On the contrary, a participant (35–44, Woman, N1) questioned the value of the vaccine as they kept “(…) hearing about new variants coming and then new restrictions and lockdown and everything (…)”. This led them to doubt the need for vaccines, since in their perspective was that “even those who are double vaccinated still have to keep all the rules of social distancing (…)”.

A lack of understanding of how vaccines work, which may have resulted in a perception of severe vaccine side effects, is evident by the following quote:

*“I don't want something in my body that could change my DNA or my genetic material. That is very important to me as a woman who can produce offspring. I don't want my genetics, my DNA [messed] with. That's important to me.” – 25-34, Woman, FNMI7*

Perceived political agendas behind vaccination campaigns were seen as shaping individuals’ by undermining the level of trust in the vaccines. Relatedly, trust in the government was seen as an influencing factor of VH, with lower trust in the government seemingly increasing VH and higher trust in the government seemingly decreasing VH. For instance, despite receiving both vaccine doses, a participant (25–34, Woman, GP10) disclosed with us the process of “(…) going back and forth because [they] don't really trust the government in a lot of things”. Their delay in receiving a vaccine demonstrates their hesitancy.

### Vaccine compliance vs vaccine acceptance across the general population

While most participants were vaccinated, VH was still present, with vaccine hesitant participants only getting vaccinated either because it was mandated of them or because they felt pressured by their families to do so. Among those who were against vaccine mandates, we identified the following key themes:

- Lack of autonomy;
- Government control, and
- Violation of citizens’ rights and freedoms.

For instance, a participant (18–24, Man, LI7) describes the consequences of not following the mandate (e.g., receiving a fine, not allowed to enter certain establishments) as a form of “punishment”. For this participant “(…) to punish somebody for not taking something that they know is a risk to them with a hundred percent certainty that there's that possibility, it's morally and ethically wrong”. This quote illustrates the perspective that vaccine mandates run counter to freedom of choice and autonomy.

In contrast, some participants acknowledged that mandates are important and necessary in some contexts, such as grocery stores, medical offices/hospitals, and the military. Responsibility for others within the community (social responsibility) versus personal freedoms (personal choice) was also discussed in relation to vaccine mandates.

*“I can remember when I went to school, and the big emphasis was on positioning like a scale of rights on one side and responsibilities on the other. If you had the right, you had a responsibility. If you had a responsibility, then you should have an equal weight. But now people seem to be so focused on the right, they forget about responsibility.” – 75*+*, Woman, LI4*

### Contextual factors surrounding equity-deserving groups’ attitudes and beliefs

In the section that follows, we present contextual factors surrounding equity-deserving groups’ attitudes and beliefs that are more unique to these groups, beyond those experienced across the general population. Equity-deserving groups described VH in terms of the following key themes:

- Fatalistic beliefs in divine will and predeterminism;
- Rapid development and production of COVID-19 vaccines;
- Experiences with the healthcare system;
- Distrust of the government;
- Personal liberty and vaccine mandates, and
- Support for government.

- Fatalistic beliefs in divine will and predeterminism

Unique to our participants who identified as FNMI were discussions of fatalistic beliefs in divine will/predeterminism driving hesitancy. For example, a participant (35–44, Woman, FNMI4) discussed COVID-19 infections as “mother nature's way of cleansing things”. For this subgroup, contextual factors such as religious beliefs or fate were identified as playing an important role in how individuals perceived the need for vaccines or how they perceived the risk of COVID-19. For example, another participant highlighted the process of actively taking action to protect oneself with vaccines, and how perhaps one should just accept one’s fate.

*“I've just about had it. It's like, I had to take one for pneumonia. I had to take one for the flu, had to take it because of my lungs. And I thought I just had it. Maybe my time is my time” – 55-64, Woman, FNMI6*

- Rapid development and production of COVID-19 vaccines

Relating to risk, the lack of testing and speed of production of the vaccines was associated with doubts about their safety and efficacy across FNMI, Black Canadians and LGBT2SQ + participants.

This was identified as a prominent factor impacting the VH of equity-deserving groups. For instance, the following two quotes illustrate that individuals were worried about how fast COVID-19 vaccines were developed, leading them to question the safety of the vaccine and risk to their health and well-being:

*“I was a little hesitant, just because of the mRNA. I mean, they say it's been around forever. But, at the same time, I mean, I think we all should've been able to sign an informed consent that this was experimental, because, I mean, even the guy who made the mRNA said, ‘Until you've done 10 years of human trials, it's still experimental.’ I was a little worried because of that. I didn't want my son to take it.” – 35-44, Woman, FNMI4*

*“To me, it's basically just like pharmaceutical and medical marketing, trying to push a product because they feel it's going to be effective. They don't know if it's going to be effective. They're just going off of numbers and statistics and research, and their own research.” – 35-44, Man, BC3*

As a consequence, one participant (35–44, Woman, LGBT2SQ + 2) shared not wanting to be “(…) the first to get a vaccine (…)”. To this same participant, “[the vaccine] was just very much in a trial phase and from [their] experience working with trials, [they] felt very uncomfortable participating in something that [they were] being tested on.”

- Experiences with the healthcare system

The previous quotes on hesitancy due to the rapid development and production of COVID-19 vaccines also relate to the subgroups’ experiences with the healthcare system. Members of the LGBT2SQ + community, as well as Black Canadians, expressed their distrust of the healthcare system as being a key factor impacting their decision-making regarding COVID-19 vaccines. For instance, a member of the LGBT2SQ + community explained how members of their community worried they would be treated differently by the medical community.

*“When you identify as part of the queer the community, I think that the first thing is that there's always a little part of your brain when you're encountering a health care provider, especially someone that you're not familiar with, that they might have personal biases or they might be personally uncomfortable, for whatever reason (...) I think that's a negative because it's like an extra step or an extra barrier or an extra condition to how we access or receive by health care settings.” – 25-34, Man, LGBT2SQ*+ *4*

Relatedly, a participant from the Black community (35–44, Man, BC3) explained how “everybody's genetic makeup is different. Someone may get the vaccine and be fine. Someone may get the vaccine and be very ill from it. It's not a guarantee”. However, this participant also explained that despite this, “(…) healthcare officials would portray [COVID-19 vaccines] as a beneficial thing to do for yourself, your household, your colleagues, your society, your community”. They further explained that this stance by the healthcare officials is not adequate since “they're not you”, and thus “they can't really say what's in your best interest”. This shared perspective highlights a lack of trust in the ability of healthcare professionals to understand and advocate for the unique needs of their patients.

- Distrust of the government

Distrust of the government, as it relates to COVID-19 VH, was expressed across several groups. For instance, below, an LGBT2SQ + participant discusses their perception of the government as self-serving, as opposed to serving the population and at-risk communities such as the LGBT2SQ + community.

*“I have little to no trust in my government. I feel that the government is the powers that be, or, for example, Trudeau, are simply symbols of many other moving parts and many of these moving parts are self-serving. (…) And I don't believe that many of the promises and agreements that it makes and dealings that it does, are not always in the best interest of the community.” – 25-34, Woman, LGBT2SQ*+ *5*

This aligns with experiences from low-income participants, where they discussed feeling misunderstood and unvalued by the government. They further expressed how the definition of being low-income alone is problematic and not representative of the diversity within this subgroup. For instance, one participant (18–24, Man, LI7) stated that they believed that “(…) [the government] should redefine what they mean by low income”. The same participant explained that “things have changed, things have increased, prices have changed”, suggesting that perhaps the definition is fluid and must be revisited to ensure the needs of the population are being met. A greater understanding of the experiences of low-income Canadians was seen as beneficial to helping the government better serve individuals within this community and help them feel heard and valued.

Participants also talked about feelings of defiance. For example, a following participant (55–64, Woman, FNMI6), discussed their feelings towards government in relation to COVID-19 vaccine mandates:

*“I think it's just defiance. I've had it with the whole health care telling us what we have to do now. And I'm so offended. (…) When did somebody think that because they had an outbreak of something, it's okay to take away people's rights or have them forced to give some of your medical information? I don't understand why that is so acceptable now. I get it. We're in a panic. I get we are going through something bad. But we've had bad things happen before.” – 55-64, Woman, FNMI6*

- Personal liberty and vaccine mandates

Another prominent factor associated with VH was objection to COVID-19 vaccine mandates. Black Canadians perceived this as a violation of their ability to advocate for themselves and as a barrier to Black Canadians’ own decisions. For example, one participant (25–34, Woman, BC4) shared their lack of choice in their vaccination status, sharing that they were forced to get it “so that's why [they are] going to eventually do it, but [they] would prefer not to”. They then followed this statement by stating that “it doesn't seem like [they] have much to say about it at this point”, which seems to be an expression of lack of choice.

Vaccine mandates were also described by this subgroup as not beneficial to the general population and as a violation of the government’s responsibility to protect the population. Some low-income participants who reported being hesitant about the COVID-19 vaccine felt they had no choice in whether they received the vaccine since the alternative would be unemployment, which may suggest a form of resentful acceptance or rather, adherence without acceptance. For instance, a participant (55–64, Woman, LI2) talked about having to take “a second job to help with [their] financial situation”, however, that job “falls under that new umbrella that Doug [premier of Ontario] put out this week”, and as consequence they now “have to get vaccinated, or [they] don't work”. They went on to state their lack of choice in accepting the vaccine, “if [they] don't work, then [they] don't pay my bills”. Another participant shared their discontent with vaccine mandates, challenging the government decision to dismiss healthcare workers refusing the vaccine by stating:

*“(…) When it comes to the point that you're firing... essentially firing. They call it leave without pay, but they're basically off the job, 290 healthcare workers, healthcare workers who last year were being heroes for treating people with this and risking their lives at a guaranteed risk to combat this virus, and then a year later just to get rid of them and say, yeah, we don't value you anymore because you're not going along with our narrative, it's not good.” – 18-24, Man, LI7*

Additionally, and in line with earlier data regarding personal liberty as a factor driving the rejection of vaccination, the following quote shows the opinion of a participant who identified as FNMI arguing that by being in unceded territory, they are not within Canadian jurisdiction and, as such, should not have to abide by rules put in place by the government.

*“So, [not receiving] the booster would be out of defiance. It's up to me. It's not up to you whether I get it. It's I don't want to put it on a piece of paper and hand it to border security going across the border. It's not right. It's not my border. (…) That's the Canadian border. It's not my border. So, I have different views on some things.” – 55-64, Woman, FNMI6*

- Support for government

Contrary to previous groups, newcomers stood out by being generally very trusting of the government and accepting of COVID-19 vaccination. For instance, a participant (25–34, Man, N9) stated “any person coming to Canada will follow everything they need to follow to come here because [they, as newcomers] want to be here. If [newcomers] had to get a vaccine, they will get it. If [newcomers] had to quarantine, they will quarantine. [Newcomers] will respect the rules.” Another participant (18–24, Woman, N8), provided greater insight into this phenomenon, by explaining that newcomers “(…) don't want a situation where they have to get deported to their country just because of a pandemic, kind of a thing”, and how newcomers “don't want their dream of a better life to be thrown away”.

Being new to Canada and being offered the opportunity to immigrate and start a new life in a new country seem to be associated with vaccine acceptance, seemingly arising from feelings of both gratitude and fear of deportation, as demonstrated in the quote below:

*“We are arriving at a new country, so in my case, I want to learn about the country, and I want to follow all the rules. So I don't know if all newcomers are the same, but if they think like I think, they are easy because they want to make the thing correctly in this new country.” – 35-44, Woman, N7*

## Discussion

This study aimed to investigate contextual factors contributing to COVID-19 VH across equity-deserving populations in Canada. Our findings speak to the nature of COVID-19 hesitancy, as observed during pre-pandemic vaccine campaigns and during the early stages of vaccine availability. In many ways data are consistent with the existing literature on COVID-19 vaccine hesitancy [16, 17, 37–40]. From this, we can conclude that equity-deserving groups share many of the same beliefs/attitudes that fuel VH in the general population. Our novel data, however, contribute to a growing body of research acknowledging the contextual factors driving vaccine-related attitudes and beliefs, and the historical, political, and sociocultural factors impacting VH in equity-deserving populations. These contextual factors require consideration and response by government and should inform efforts towards meaningful engagement with community as a starting point to promote the confident acceptance of novel vaccines among equity-deserving groups within Canada. We discuss key findings as they relate to each subgroup below.

### Contextual factors for participants identifying as FNMI: themes of fatalistic beliefs in divine will/predeterminism, feelings of defiance toward government mandates, perceptions relating to experimental vaccines and informed consent, history of oppression and discrimination, and distrust of the healthcare system

Participants identifying as FNMI discussed topics relating to themes of fatalistic beliefs in divine will/predeterminism as reasons not to accept the COVID-19 vaccine. Without expertise in this area, we suggest further research that engages FNMI communities to better understand and work to support vaccine acceptance in a manner that respects and accounts for these beliefs as a next step forward. Data also points to the importance of taking action to acknowledge and respond to feelings of defiance toward government mandates in association with VH. Historical experiences of oppression and cultural genocide across generations of FNMI communities may be exacerbated by the Government of Canada still failing to meet the needs of Indigenous People, as they experience barriers to adequate health care, healthy food, clean water supply, and experience issues such as overcrowded housing, homelessness, and high levels of incarceration [41]. Relatedly, participants identifying as FNMI also discussed experimental vaccines and informed consent as reasons for VH. While the general concern that vaccines can have severe side effects exists among the general population, the history of medical experimentation, especially among children, that occurred on reserves and in residential schools in Canada [42], may explain why participants expressed hesitancy toward the vaccine, questioning its ‘experimental’ nature and concerns regarding informed consent. This might also relate to the fact that indigenous communities were one of the priority groups for vaccines and thus one of the first population groups to be offered the vaccine [43]. This prioritization may have created or reinforced ideas of being the ‘guinea pig’ and explain a reluctance to receive COVID-19 vaccines. These historical injustices experienced by FNMI communities can in part explain the persisting opposition to government intervention and provide further weight for the need to redress historical injustices over generating more tailored promotional materials to increase vaccine acceptance. As a step forward, research has emphasized the importance of supporting Indigenous peoples’ right to self-determination, in how that may be an important step toward reducing hesitance toward the COVID-19 vaccines [44], and as such, likely novel vaccines in the future.

Contextual factors for the LGBT2SQ + community: themes of perceptions relating to experimental vaccines and vaccine safety, as history of medical harm and distrust of the healthcare system Participants that identified as LGBT2SQ + also expressed concerns regarding the lack of testing of COVID-19 vaccines and low trust in government. As with FMNI, these are commonly cited concerns regarding the COVID-19 vaccine, but the rationale for these concerns may be rooted in experiences of systemic oppression leading to a lack of trust and VH [45]. For example, society continues to privilege heterosexuality, which perpetuates the stigma and inequality negatively impacting gay and bisexual men [46]. Furthermore, social marginalization and sexual health inequalities are found to contribute to behaviors such as unwillingness to seek and receive needed and adequate services and medical care [47]. Indeed, historical and ongoing medical trauma, including misgendering and perceived emotional violence, have been found to be barriers to trust in the medical system and consequently to the uptake of COVID-19 vaccines [48]. For example, Twitter data collected during COVID-19 found that posters used to promote COVID-19 vaccination were viewed as stigmatizing, akin to the promotion of preexposure prophylaxis [18], a medication for people who do not have HIV but are at higher risk of exposure to it, via sex or injection-drug use [49]. This showcases how a legacy of harm caused by healthcare institutions contributes to COVID-19 VH in this community of individuals [18]. In addition, it was only in 1969 that Prime Minister Pierre Trudeau’s proposed amendments to the Criminal Code permitted the decriminalization of homosexuality in Canada [50]. The unique relationship between LGBT2SQ + and the government may help with the understanding of why some individuals in this subgroup are hesitant upon being asked to receive a COVID-19 vaccine. Broken trust, from a long history of oppression and persecution, needs to be rebuilt for greater adherence to vaccine mandates.

### Contextual factors for Black Canadians: themes of perceived lack of autonomy, history of oppression and discrimination, and distrust of the healthcare system

Black participants in our research cited concerns regarding COVID-19 that largely reflect those of the general population, namely, distrusting healthcare providers/the healthcare system, rejection of vaccine mandates, and concerns about vaccine safety. However, the contextual factors discussed provide novel insight. In relation to vaccine mandates, participants described the denial of their rights and feeling that decisions were being made for members of the Black community and hesitancy related to medical distrust. These findings have been reported elsewhere in the Canadian context [51] and support calls to action Black-led partnerships between health care and stakeholders with existing trusted relationships in the community to increase confidence in SARS-CoV-2 vaccination in Black communities. This will also be an important consideration moving forward in the promotion of vaccination more broadly, particularly with novel vaccines to address emerging infectious diseases.

### Contextual factors for low-income Canadians: themes of feeling misunderstood, distrust in the government, financial stress and unemployment threat, autonomy, and personal liberty

Low-income individuals in this study discussed feeling misunderstood by the government, which impacted their acceptance of the COVID-19 vaccines. Participants justified these feelings by sharing how they believe that those in power, namely, political leaders, have not experienced some of what the low-income community has experienced, creating a sense of disconnect between the two. Some of the distrust in authority and concerns of conflict of interest in this subgroup may arise from failures in a formal representative democracy of low-income individuals, which leads to situations where wealthy peoples’ opinions carry more weight than the opinions of the poor [52]. Representation is important because the preference in policies for higher income individuals may be different than that of the low-income subgroup [52]. Lack of representation and communication between local government and members of this subgroup can greatly impact the trust that low-income individuals have in public health and government, both of which can lead to VH. Distrust in the government and feeling misunderstood by political leaders raises questions about the target demographic that benefits most from recommendations and mandates set about in response to COVID-19.

Low-income participants also cited concerns regarding the lack of prioritization of employment over vaccination status. The threat of unemployment is more salient for low-income individuals, since low-income individuals often do not have a safety net and experience barriers in access to quality food, hosing care, and safety, as well as experience financial stress and poor mental health and more often engage in risky behaviors [53]. In the context of vaccine mandates, losing their job and further aggravating their financial stress puts individuals with lower incomes in a particularly unique place, where they adhere to COVID-19 vaccination, albeit begrudgingly. These factors contributed to discontent and anger toward the government, which may further exacerbate VH, and discussed defiance relating to the COVID-19 vaccines. Given that VH has been found to greatly affect individuals with lower socioeconomic status (e.g., lower education or income levels) [17], it is important that greater efforts are made to support this subgroup.

### Contextual factors for newcomer to Canada: themes of support the government, and acceptance of vaccines

Newcomer participants were found to be generally accepting of the COVID-19 vaccine, a finding that is inconsistent with research suggesting that the odds of VH among immigrants in Canada are approximately two times greater than their Canadian‐born counterparts [16]. It is possible that this finding reflects the newcomer status – as one of perceived vulnerability – as our participants cited explanations for acceptance as they related to following the ‘rules’, doing what is asked of them, and not wanting to ‘throw away’ their new lives for not doing so. This is consistent with recent published work (e.g., see) [54]). It will be important to move forward to support newcomers to confidently accept vaccines and health information more broadly based on informed choice as opposed to perceived fear of punitive repercussions.

### Eroded trust in the government and public health across equity-deserving subgroups

Underlying our themes and impacting the level of acceptance of novel vaccines across groups, appears to be an issue of eroding trust, particularly trust towards the government and public health. Previous research has found trust to be a critical factor impacting vaccine decision making, and thus VH [55, 56]. However, it has been suggested that a gap exists in the understanding of the process through which trust can be lost [57]. It is important to acknowledge that concepts of trust, mistrust and distrust are interrelated with one another, and that individuals experiencing VH may be able to change if the experiences they have with healthcare institutions and governments change [57]. While it is possible for healthcare professionals to “partially repair the severed relationship” [57], this is simply a band-aid solution. Our findings provide some insight into contextual influences that may explain the process of losing trust across equity-deserving groups. However, it is important to acknowledge that trust as a concept is quite complex, and that trust may be more difficult to (re)build if it has been eroding over many generations as opposed to being challenged during the COVID-19 pandemic. Future research should keep exploring the process of losing trust and the role it has on VH, across different vaccines and population groups. Identifying and understanding contextual factors driving vaccine-related attitudes and beliefs, and the historical, political, and sociocultural factors impacting VH, has the potential to support trust between equity-deserving groups and the Canadian government, and may support the development of interventions to increase the confident acceptance of vaccines, and particularly novel vaccines.

### Going beyond tailored promotion efforts

Our findings suggest that governments must work with equity-deserving groups to address vaccine-related anxieties and worries by acknowledging and responding to their needs. This approach may take different forms and will vary depending on the target population. While we did not co-develop our work with affected communities and acknowledge this as a limitation, our findings provide a foundation upon which to engage with communities and develop human-centered design strategies, such as co-creation, co-design, and co-production efforts, focused on VH across equity-deserving groups [58]. For example, previous work done in Montreal, Canada, supports the benefits of these strategies to address VH among children and youth [59]. Our work may help inform the design of strategies that can be used by researchers, government agencies, and policymakers to engage equity-deserving groups, taking into consideration the contextual factors shaping behaviors. A first step may be to acknowledge historical relationships with government and public health, respond to injustices of the past, and demonstrate trustworthiness and respect for including the voices of representatives of equity-deserving populations. Successful engagement with equity-deserving groups to promote vaccine acceptance, that take into consideration of the unique identities, experiences, and needs of underserved populations exist [60]. This work might be replicated within Canada with a focus on individuals who identify as First Nations, Métis, Inuit, LGBT2SQ + , low-income and/or Black Canadians.

### Limitations

When collecting demographic data for participants involved in the study, level of education was not considered, despite being a key influencing factor of VH. While some sources interchangeably use education or level of income as a good indicator of SES, it is possible that data on low levels of education could have provided further insight into some of the themes presented. Political affiliation as a participant characteristic has also been suggested to be a strong influencing factor related to VH and should be a consideration in research investigating the acceptance of novel vaccines in the future. Our recruitment via Leger precluded us from obtaining perspectives of equity-deserving groups marginalized based on language (non-English or French speaking), those with the inability to be recruited or participate due to literacy or access to technology or from indigenous peoples living on reserve. Lastly, we were not able to ensure congruence between interviewer and interviewee for all subgroups of focus. We acknowledge this is the limitation in both the collection, analysis, and interpretation of data. Our team will prioritize representation of marginalised populations, who understand the unique contexts of these communities in Canada, in future research.

## Conclusions

VH is complex and multifaceted. This study highlights demographic and contextual factors associated with COVID-19 VH that are unique to equity-deserving groups within Canada. While the data regarding hesitancy largely mirror concerns reported in the vast body of literature citing rationale for COVID-19 hesitancy in high-income countries, the contextual factors related to historic and ongoing oppression point to the need for wider systemic change, over or in conjunction with tailored promotional materials. Herein, we identified novel themes – e.g., fatalistic beliefs in divine will/predeterminism – that demonstrate a need for greater engagement with the community to better understand and support promotion efforts that do not run counter to belief systems. Our data provide government agencies and policymakers with an overview of the contextual factors influencing VH among equity-deserving groups that relate to unmet needs that should be addressed *before* we can expect attitudes and behaviours to change. Government and health officials might act on these findings by working with communities to co-design/co-produce efforts to address VH; going beyond simply tailoring promotional campaigns. As we “exit” the COVID-19 pandemic and see the emergence of novel infectious diseases and related vaccines, hesitancy in equity-deserving groups should continue to be a priority for public health across Canada. We focus here on historical and political factors that are and should continue to be redressed to promote the confident acceptance of health promotion efforts now and moving forward.

## Data Availability

Data are not available as it is a requirement of the ethics committee that data remain confidential.

## Abbreviations

VH: Vaccine Hesitancy
GP: General Population
BC: Black Canadians
LGBT2SQ +: Lesbian, Gay, Bisexual, Transgender, Two-Spirit, Queer, and additional sexual orientations, and gender identities under the LGBT2SQ + umbrella
FNMI: First Nations, Métis, and Inuit
LI: Low Income
N: Newcomer
NL: Newfoundland and Labrador
NS: Nova Scotia
ON: Ontario
AB: Alberta
BIPOC: Black, Indigenous, People of Color
